# ﻿Three new species of *Ochrolechia* (Ochrolechiaceae, Pertusariales) from Guizhou Province, China

**DOI:** 10.3897/mycokeys.126.168652

**Published:** 2025-12-02

**Authors:** Weiwei Zheng, Linzhi He, Heyun Bo, Rajesh Jeewon, Ruvishika S. Jayawardena, Yuxian Wang, Jie Wang, Shaobin Fu, Qingfeng Meng

**Affiliations:** 1 School of Pharmacy, Zunyi Medical University, Zunyi, Guizhou Province 563000, China; 2 Department of Health Sciences, Faculty of Science, University of Mauritius, Reduit 80837, Mauritius; 3 Center of Excellence in Fungal Research, Mae Fah Luang University, Chiang Rai 57100, Thailand; 4 School of Science, Mae Fah Luang University, Chiang Rai 57100, Thailand; 5 School of Public Health, Zunyi Medical University, Zunyi, Guizhou Province 563000, China

**Keywords:** Lichen, morphology, phylogeny, taxonomy, three new species

## Abstract

Based on morphological, chemical and phylogenetic analyses, three species of *Ochrolechia* collected from Guizhou, China, are described as new to science and named *Ochrolechia guizhouensis*, *O. kuankuoshuiensis* and *O. leigongshanensis*. Phylogenetic trees, based on ITS and mtSSU loci, were constructed using Maximum Likelihood analysis (ML) and Bayesian Inference (BI) methods. *Ochrolechia guizhouensis* and *O. akagiensis* are positioned close to each other in the phylogenetic tree. *Ochrolechia guizhouensis* is morphologically characterised by its apothecia which feature rugose to rosulate, epruinose discs resembling a floral structure. Chemically, the apothecia contain gyrophoric acid, lecanoric acid and lichesterinic acid, while the thallus contains only gyrophoric acid and lecanoric acid. Phylogenetic analysis reveals that *Ochrolechia kuankuoshuiensis* is closely related to *O. parellula* and is distinguished by its light yellow, epruinose, deeply rugose apothecial discs, the production of gyrophoric acid and lecanoric acid and the largest ascospores known in the genus. *Ochrolechia leigongshanensis* forms an isolated phylogenetic branch which is characterised by a soraliate thallus and apothecia with a ring of smooth, salmon-pink tissue on the inner margin. The discs are epruinose to lightly pruinose and plane. The thallus contains gyrophoric acid, lecanoric acid and a trace of atranorin, while the apothecia contain only gyrophoric acid and lecanoric acid. Additionally, *O. subrosella* and *O. longispora* were successfully sequenced for the first time and represent the first records from Guizhou Province. We found one specimen, which phylogenetically groups with *O. trochophora*, but is characterised by the presence of soralia, indicating that *O. trochophora* might be morphologically more variable than previously thought. Colour photographs are provided for all the above-mentioned species.

## ﻿Introduction

Massalongo (1852) established the crustose lichen genus *Ochrolechia* by transferring six taxa from *Lecanora* to this genus. *Ochrolechia* is characterised by a crustose thallus that may be continuous or fissured and varies from thin to thick, with a pale white to grey or even yellowish coloration. Soredia are often present in some species and a few taxa also produce isidia (Tonsberg 1992; Elix 2007). The apothecia are typically large, with a disc that may be epruinose or pruinose; a strongly amyloid hymenium; paraphyses are slender, branched and interwoven and asci generally contain four to eight hyaline, simple, thin-walled ascospores (Schmitt and Lumbsch 2004; Ren 2017).

The taxonomy of *Ochrolechia* has undergone significant revisions since its establishment. Reinke (1894) initially placed *Ochrolechia* in the Pertusariaceae within Parmeliales. Choisy (1949) later maintained its familial placement, but transferred it to Pertusariales. Subsequently, Verseghy (1962) proposed a classification for *Ochrolechia*, dividing 59 species into five informal groups: Geminiparae, Parella, Upsaliensis, Tartarella and Harmandi. Advances in molecular phylogenetics later prompted a re-assessment of its systematic position, leading to the establishment of the family Ochrolechiaceae to accommodate the genus (Schmitt et al. 2006). Ochrolechiaceae is currently a monogeneric family, comprising only *Ochrolechia* and is placed within Pertusariales (Hyde et al. 2024).

In a systematic revision of the Chinese species of *Ochrolechia*, Ren (2017) recognised 20 taxa. The genus is mainly distributed in the south-western provinces, particularly Yunnan and Sichuan, with scattered records from Fujian, Gansu, Shaanxi, Anhui and Hunan, typically occurring at elevations above 1,500 m. In that study, four new species (*O. alticola*, *O. mexicana*, *O. lijiangensis*, *O. rugomarginata*) were described, four taxa (*O. arborea*, *O. mahluensis*, *O. szatalaënsi*, O. trochophora var. pruinirosella) were newly recorded for China and an identification key to all 20 taxa was provided. Subsequently, *O. parellula* was added to the Chinese mycobiota by Fu et al. (2022).

Chemical compounds of lichens, particularly secondary metabolites, such as depsides, anthraquinones and triterpenoids in lichens, have long been recognised as reliable taxonomic markers in lichen identification (Culberson 1969; Huneck and Yoshimura 1996). In *Ochrolechia*, the occurrence and distribution of diagnostic substances (e.g. gyrophoric acid and lecanoric acid) have been widely used for species delimitation and identification. Amongst these, gyrophoric acid is the predominant metabolite. Other depsides that produce a C + red reaction, such as olivetoric acid and lecanoric acid, are typically only in combination with gyrophoric acid (Brodo 1991).

In this study, *Ochrolechia* specimens collected from Guizhou Province were identified using an integrative approach combining morphology, phylogeny and chemistry.

## ﻿Materials and methods

### ﻿Sample collection and morphological examination

The specimens (KKS83, KKS150-2) were collected from Kuankuoshui National Nature Reserve and others (LGS40, LGS176, LGS186, LGS205, LGS213-2, LGS219) from Leigongshan National Nature Reserve, Guizhou Province, China. Macro-morphological characteristics were observed using a stereoscopic microscope (XTL-3B, Coic). The ascomatal sections were manually prepared as temporary mounts and observed under a compound microscope (Olympus BX53), with photographic documentation obtained using an attached digital camera (Olympus DP72). Measurements were conducted using the Image Framework software (Tarosoft, version 0.9.7). The dimensions of asci and ascospores were recorded (for n ≥ 10) and presented as: (min-) [x̄ – SD] – [x̄ + SD] (max), where “min” and “max” represent extreme values, x̄ is the arithmetic mean and SD is the standard deviation (Meng et al. 2025). Unless otherwise specified, all measurements were taken from water-mounted sections, following the procedures outlined by Senanayake et al. (2020). Holotype specimens are deposited in the Lichen Herbarium of Kunming Institute of Botany (KUN-L), Chinese Academy of Science, Yunnan, China.

### ﻿Determination of secondary metabolites

Secondary metabolites were examined using chemical spot tests under a stereoscopic microscope and thin-layer chromatography (TLC) for the thallus and apothecia, respectively. The spot tests included 10% aqueous potassium hydroxide (K), saturated aqueous calcium hypochlorite (C) and a sequential application of K followed by C (KC). TLC was performed using the following solvent systems: A (toluene: dioxane: acetic acid = 180: 60: 8, v/v), B’ (hexane: methyl tert-butyl ether: formic acid = 140: 72: 18, v/v), and C (toluene: acetic acid = 200: 30, v/v) (Culberson and Kristinsson 1970; Culberson 1972; Wei and Qiu 1998; Orange et al. 2001). Metabolites were identified by comparison with reference compounds in *Lethariella cladonioides*, which contains atranorin and norstictic acid (Dou et al. 2024).

### ﻿Extraction and amplification of DNA

Genomic DNA was extracted from the specimens using a Fungal Genomic DNA Kit (Sangon Biotech, Shanghai) following the manufacturer’s instructions. The internal transcribed spacer region of the nuclear ribosomal DNA (ITS) was amplified using the primers pairs ITS1f/ITS4 (White et al. 1990) and the mitochondrial small subunit ribosomal RNA gene (mtSSU) was amplified using the primer pairs mrSSU1/mrSSU3r (Zoller et al. 1999). Polymerase chain reactions (PCR) were performed using a Mastercycler (Bio-RAD T-100) in a 20-µl reaction volume consisting of 10 µl of 2× Mix (Solarbio, dNTPs Mix), 6.4 µl of double-distilled water (ddH_2_O), 2 µl of the DNA template and 0.8 µl of each primer. The PCR amplification reaction procedures are listed in Table 1.

**Table 1.** Primer pairs and the parameters for PCR.

Locus	Primers	Initial Denaturation	Denaturation	Annealing	Elongation	Final Extension	Hold
ITS	ITS1f/ITS4	95 °C/3 min	95 °C/30 s	55 °C/30 s	72 °C/90 s	72 °C/10 min	4 °C/+∞
			38 cycles				
mtSSU	mrSSU1/mrSSU3r	95 °C/3 min	95 °C/30 s	53 °C/30 s	72 °C/1 min	72 °C/10 min	4 °C/+∞
			38 cycles				

### ﻿Phylogenetic analysis

The quality of the electropherograms was checked using BioEdit software. Forward and reverse sequences were assembled using the ContigExpress software (New York, USA) and the concatenated sequences were subjected to BLASTn searches in NCBI (www.ncbi.nlm.nih.gov/) for preliminary identification. The BLAST results of all newly-generated sequences indicated that these samples belong to *Ochrolechia*. The related taxa were retrieved from GenBank and combined with newly-generated sequences to conduct a phylogenetic analysis (Table 2). Two vouchers of *Trapelia coarctata* were selected as the outgroup (Park et al. 2019). Multiple alignments were performed using online MAFFT version 7 (http://mafft.cbrc.jp/alignment/-server), with default settings and trimmed using trimAl v1. (Capella-Gutiérrez et al. 2009; Katoh and Standley 2013). The ITS and mtSSU were merged in SequenceMatrix-Windows 1.7.8 and the aligned FASTA files were subsequently converted to NEXUS format using AliView v.1.27 for Bayesian Inference analysis (Glez-Peña et al. 2010). Twenty-two common DNA substitution models with rate heterogeneity were tested by ModelFinder (Kalyaanamoorthy et al. 2017). The best-fit model for each gene region, as determined by the Bayesian Information Criterion (BIC), is as follows: ITS: TIM2+F+I+G4, mtSSU: TVMu+F+R2. The resulting sequences were imported into the CIPRES Science Gateway (www.phylo.org/portal2/home.action) for RAxML analysis (Vaidya et al. 2011) and MrBayes analysis. The ML analysis was conducted with the RAxML-HPC2 tool on XSEDE (8.2.12) employing a GTRGAMMA approximation with a rapid bootstrap analysis of 1000 replicates (Stamatakis 2014). The Bayesian Inference phylogenies were inferred using MrBayes v.3.2.7a on XSEDE (Ronquist et al. 2012) (2 parallel runs, 10,000,000 generations), in which the initial 25% of sampled data were discarded as burn-in phase. The phylogenetic trees were visualised in FigTree v.1.4.4 and edited in Adobe Illustrator CC 2019; the resulting tree only displays ML bootstrap support values ≥ 70 and BI posterior probabilities ≥ 0.90, respectively. New species are identified, based on recommendations outlined by Jeewon and Hyde (2016).

**Table 2.** Taxa used in this study and their GenBank accession numbers, “NA” indicates unavailable sequence.

Species	Culture/voucher	ITS	mtSSU
*Ochrolechia akagiensis*	Hara Kojiro:0007	LC533077	NA
*O. alaskana*	TROM_L_60552	MK811874	NA
*O. alboflavescens*	PRA-Vondrak22558	OQ717978	OQ646357
*O. alboflavescens*	O-L-201276	MK812244	NA
*O. androgyna*	PRA-Vondrak23666	OQ717979	OQ646358
*O. androgyna*	PRA-Vondrak23816	OQ717525	OQ646359
*O. antarctica*	Davey 27-3	NA	KX499403
*O. antarctica*	Davey 21-7	NA	KX499401
*O. arborea*	PRA-Vondrak25006	OQ717981	OQ646360
*O. austroamericana*	Flakus 21197	NA	KX499404
*O. bahusiensis*	PRA-Vondrak22578	OQ717983	OQ646362
*O. bahusiensis*	17950	MN387044	NA
*O. balcanica*	Schmitt ESS-20968	NA	AF329170
*O. frigida*	ERCH: HS 51	OR687726	NA
*O. frigida*	P168	KR017062	KR017339
***O. guizhouensis***	**LGS219**	**PV930363**	**PV930371**
*O. gowardii*	O-L-200100	MK812175	NA
*O. gowardii*	O-L-200283	MK812101	NA
*O. incarnata*	Ertz 10572	MH485185	NA
*O. incarnata*	GLM25533	MH485183	NA
*O. juvenalis*	AFTOL-ID 374	HQ650719	KJ766446
*O. kerguelensis*	Ertz 18928	NA	KX499406
*O. kerguelensis*	Ertz 18906	NA	KX499405
***O. kuankuoshuiensis***	**KKS83**	**PV930358**	**PV930366**
***O. kuankuoshuiensis***	**LGS40**	**PV930359**	**PV930367**
***O. leigongshanensis***	**LGS186**	**PV930364**	**PV930372**
***O. longispora***	**LGS205**	**PV930362**	**PV930370**
***O. longispora***	**LGS213-2**	**PV930361**	**PV930369**
*O. mahluensis*	PRA-Vondrak22577	OQ717985	OQ646363
*O. microstictoides*	PRA-Vondrak23770	OQ717987	OQ646364
*O. microstictoides*	PD032M	MW325687	NA
*O. oregonensis*	CCDB-36282-A09	OQ843356	NA
*O. oregonensis*	L-793	OQ922942	NA
*O. pallescens*	J. Malicek 10146	MK778626	MK778561
*O. pallescens*	Lumbsch, 9. Aug. 2004	NA	DQ780277
*O. parella*	K(M):202474a	MZ159609	NA
*O. parella*	Ertz 10504	MH485200	KX499409
*O. parellula*	KoLRI No.018698	KU883361	NA
*O. parellula*	KoLRI No.015662	KU883360	NA
*O. parellula*	KoLRI No.013758	KU933682	NA
*O. parellula*	KoLRI No.015650	KU883359	NA
*O. parellula*	CBM:Sakata 3456	LC489985	NA
*O. peruensis*	Lumbsch 19360c	NA	DQ780279
*O. subathallina*	L-828	OQ922944	NA
*O. subpallescens*	Lumbsch 19900a	NA	GU980978
***O. subrosella***	**LGS176**	**PV930360**	**PV930368**
*O. subviridis*	O-L-200638	MK812495	NA
*O. subviridis*	Sadowska-Des 810H13-002_G07	MH485202	NA
*O. szatalaensis*	PRA-Vondrak23372	OQ717527	NA
*O. szatalaensis*	O-L-200097	MK811817	NA
*O. tartarea*	O-L-196041	MK812341	NA
*O. tartarea*	DNA7	JN943620	NA
*O. trochophora*	AFTOL-ID 880	NA	DQ986901
*O. trochophora*	J. Vondrak 15442	MK778627	MK778562
**O. trochophora var. trochophora**	**KKS150-2**	**PV930365**	**PV930373**
*O. turneri*	PRA-Vondrak23511	OQ717989	OQ646366
*O. turneri*	PRA-JV23905	OK333002	OK465619
*O. upsaliensis*	Leavitt 18-422 BRY-C	MZ243924	NA
*O. upsaliensis*	O-L-195967	KY266960	NA
*O. xanthostoma*	Tonsberg 46121	MN483173	MN508284
*O. yasudae*	Hara Kojiro:0005	LC533076	NA
*O. yasudae*	KoLRI No.010572	KU883364	NA
*T. coarctata*	O-L-179924	MK812177	NA
*T. coarctata*	O-L-182063	MK812526	NA

## ﻿Results and discussion

### ﻿Phylogenetic analysis

**Figure 1.**
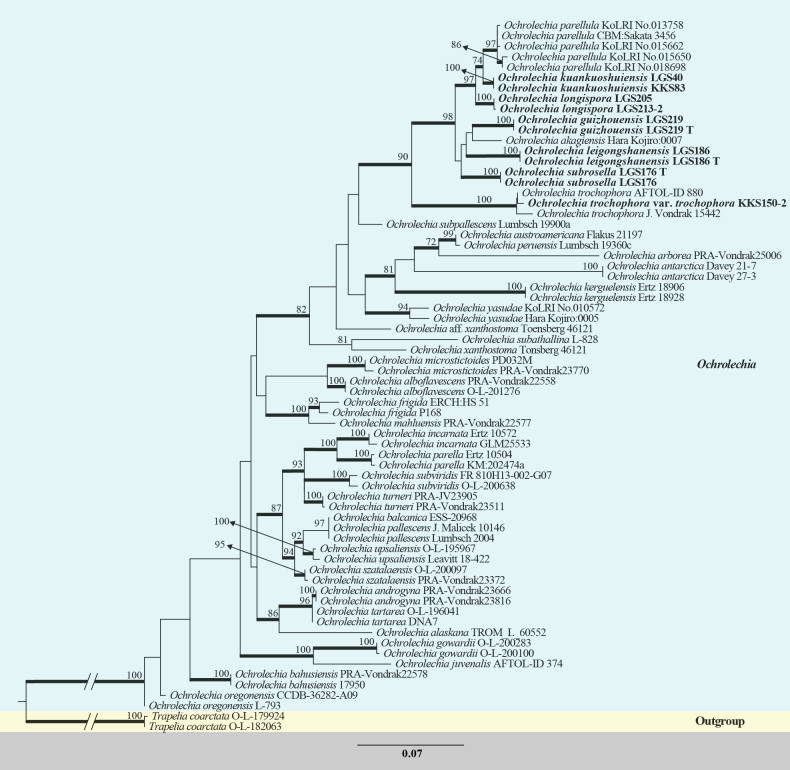
Phylogenetic relationships within the genus *Ochrolechia*. This is a RAxML analysis, based on combined ITS and mtSSU sequence data. Bootstrap support values of Maximum Likelihood (ML) ≥ 70% are labelled at corresponding positions; branches that simultaneously satisfy the criteria of ML bootstrap support ≥ 70% and Bayesian posterior probability (PP) ≥ 0.90 are displayed in bold. *T. coarctata* (O-L-179924) and *T. coarctata* (O-L-182063) were used as outgroup taxa. The newly-generated sequences are shown in bold font.

In this study, the final aligned dataset comprised 1,256 characters, including gaps (ITS:1–541, mtSSU:542–1,256). Estimated base frequencies were as follows: A = 0.2534, C = 0.2370, G = 0.2539, T = 0.2557. Substitution rates amongst nucleotides were as follows: AC = 1.3275, AG = 4.1600, AT = 2.2572, CG = 0.8425, CT = 8.1438, GT = 1.0000. The average standard deviation of split frequencies for the BI analysis reached 0.010000, indicating good convergence. The tree topologies inferred by both ML and BI analyses were manually verified and found to be highly congruent (BP/PP = 100/1 for major supported clades). The best-scoring RAxML tree, based on combined ITS and mtSSU-sequence datasets, is presented in Fig. 1.

The phylogenetic tree comprises 36 taxa of *Ochrolechia*. Analysis reveals that the species examined in this study cluster into a large clade with *O. trochophora*, *O. akagiensis* and *O. parellula* (ML Bootstrap = 90%, BI PP = 1.00). Within this clade, specimen KKS150-2 forms a distinct subclade with *O. trochophora* (ML Bootstrap = 100%, BI PP = 1.00), indicating a close phylogenetic relationship between them. *O. kuankuoshuiensis* and *O. parellula* form a sister clade (ML Bootstrap = 74%, BI PP = 0.93) positioned near *O. longispora*, suggesting a possible shared ancestry or similar evolutionary trajectory amongst these lineages. *O. guizhouensis* and *O. akagiensis* are closely related, implying a certain degree of phylogenetic affinity. However, the low support value for this relationship may reflect relatively high genetic divergence between the two species. Furthermore, *O. leigongshanensis* and *O. subrosella* each form phylogenetic lineages with low support values, indicating distant relationships to other *Ochrolechia* taxa, which may be attributable to their distinct genetic characteristics.

### ﻿Taxonomy

#### ﻿ Ochrolechia guizhouensis

Taxon classificationFungiPertusarialesOchrolechiaceae

Zheng & Meng sp. nov.

F9F6D30F-039F-5630-A60C-2BF501D98F7B

Index Fungorum: IF902063

Facesoffungi Number: FoF18024

Fig. 2

##### Remark.

*Ochrolechia guizhouensis* is characterised by apothecia with rugose to rosulate discs presenting a distinct floral morphology. Chemically, lichesterinic acid is present in the apothecia, but absent in the thallus.

**Figure 2.**
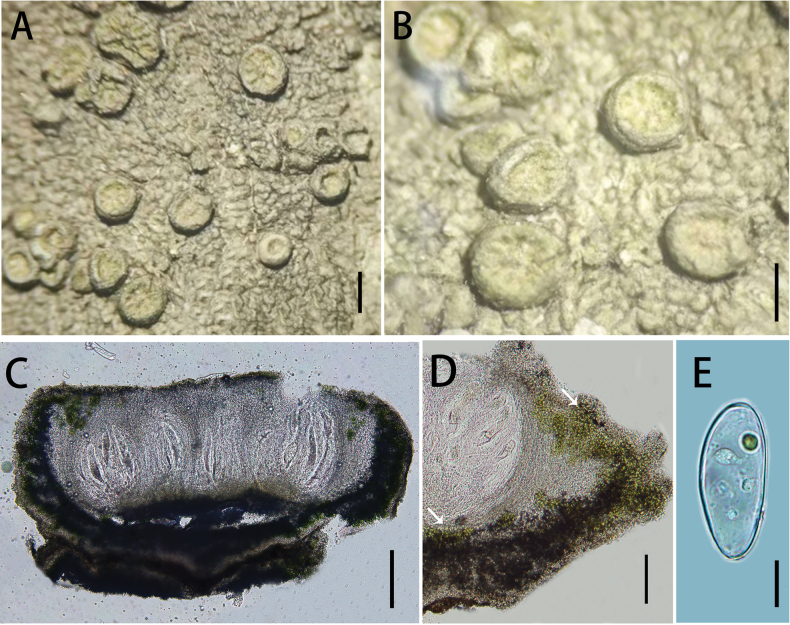
*Ochrolechia guizhouensis* (LGS219). **A, B.** Morphology of thallus and apothecia; **C.** Cross-section of the ascomata; **D.**Algae (the white arrow points to the algae); **E.** Ascospores. Scale bars: 1 mm (**A**); 0.5 mm (**B**); 200 μm (**C**); 100 μm (**D**); 20 μm (**E**).

##### Type.

China • Guizhou, Qiandongnan Prefecture, Leigongshan Nat. Res, on bark, 1698 m elev., 2023, B. Liu and Z. Yang, LGS219 (KUN-L 96618, holotype).

##### Etymology.

The species epithet refers to Guizhou Province, the type locality where the species was collected.

##### Holotype.

KUN-L 96618.

##### Description.

***Thallus*** greyish-green, thick, rugose to verruculose, dull; ***prothallus*** indistinct, ***isidia*** and ***soredia*** absent.

***Sexual morph*. *Apothecia*** frequent, scattered, sometimes crowded, sessile, round or irregular, 0.5–0.8 mm diam.; disc light yellowish-green or light brown, rugose to rosulate, epruinose; margins thick, smooth and taller than disc when young, becoming thin, verruculose and as tall as disc when older, exciple well developed even extending to the disc surface, concolorous with the thallus, shiny. Pycnidia are absent. ***Hymenium*** hyaline and colourless, 360–370 μm high, paraphyses are branched and anastomosing; epihymenium brown, 9–11 μm high; hypothecium pale brown (the colour of the epihymenium and hypothecium disappears in a 10% KOH solution), 30–40 μm high; coccoid green alga, algae forming a continuous layer in the margin and in scattered clumps below the hypothecium. ***Asci*** clavate, (269)272–284(288) × (48)49–60(62) μm (n = 10). ***Ascospores*** hyaline, aseptate, broadly ellipsoid, (57)60–78(82) × (26)28–32(34) μm (n = 31).

##### Chemistry.

Thallus contains gyrophoric acid and lecanoric acid; ascomata contain gyrophoric acid, lecanoric acid and lichesterinic acid (TLC). Thallus cortex: K + pale yellow, C + red, KC + red; medulla: C –; apothecia cortex: K + pale yellow, C + red, KC + yellow; medulla: C –; disc: C + red; thallus UV −.

##### Material examined.

China • Guizhou Province, Qiandongnan Prefecture City, Leigongshan National Nature Reserve, 26°22'43.24"N, 108°11'42.54"E, 1698 m elev., on bark, 27 October, 2023, Bo Liu and Ze Yang, LGS219 (KUN-L 96618, holotype).

##### Notes.

In the phylogenetic tree, *O. guizhouensis* (LGS219) and *O. akagiensis* (Hara Kojiro:0007) are closely related although with weak bootstrap support (< 70%). The ITS sequence divergence between them is 6.35% (33/520 bp). Morphologically, the two species differ distinctly: *O. guizhouensis* lacks isidia on the thallus and produces pale yellow, smaller apothecia (0.5–0.8 mm in diameter), whereas *O. akagiensis* bears isidia and develops pinkish, larger apothecia (0.8–1.5 mm in diameter). Chemically, the apothecia of *O. guizhouensis* contain lichesterinic acid, while the presence of this compound has not been reported in *O. akagiensis* (Park et al. 2019).

Although *Ochrolechia guizhouensis* resembles *O. trochophora* in some morphological and chemical characteristics, the two species differ notably in several features. The apothecia of *O. guizhouensis* are smaller (0.5–0.8 mm diam.), whereas those of *O. trochophora* are larger (1–3(4) mm diam.) (Brodo 1991). In addition, the apothecia of the latter lack lichesterinic acid. Phylogenetic analyses further indicate that this new species is distantly related the two *O. trochophora* vouchers (AFTOL-ID 880 and J. Vondrak 15442), supported by sequence divergences of 13.76% (67/487 bp) in the ITS region and 4.97% (35/704 bp) in the mtSSU region between *O. guizhouensis* (LGS219) and *O. trochophora* (J. Vondrak 15442).

The difference with another similar species *O. margarita*, based on the identification key of Ren (2017), is that this species has a very thin thallus and its apothecia become subglobose when mature. Moreover, the algal layer forms a continuous layer below the hymenium, containing only gyrophoric acid and a trace of lecanoric acid.

#### ﻿ Ochrolechia kuankuoshuiensis

Taxon classificationFungiPertusarialesOchrolechiaceae

Zheng & Meng sp. nov.

E2CA1B0C-178E-5946-9C23-494D7358E7C8

Index Fungorum: IF904111

Facesoffungi Number: FoF17959

Fig. 3

##### Remark.

Distinctive features of *O. kuankuoshuiensis* are the large ascospores ((90)97–116(123) × (30)33–35(36) μm) and chemistry, which is limited to gyrophoric acid and lecanoric acid.

**Figure 3.**
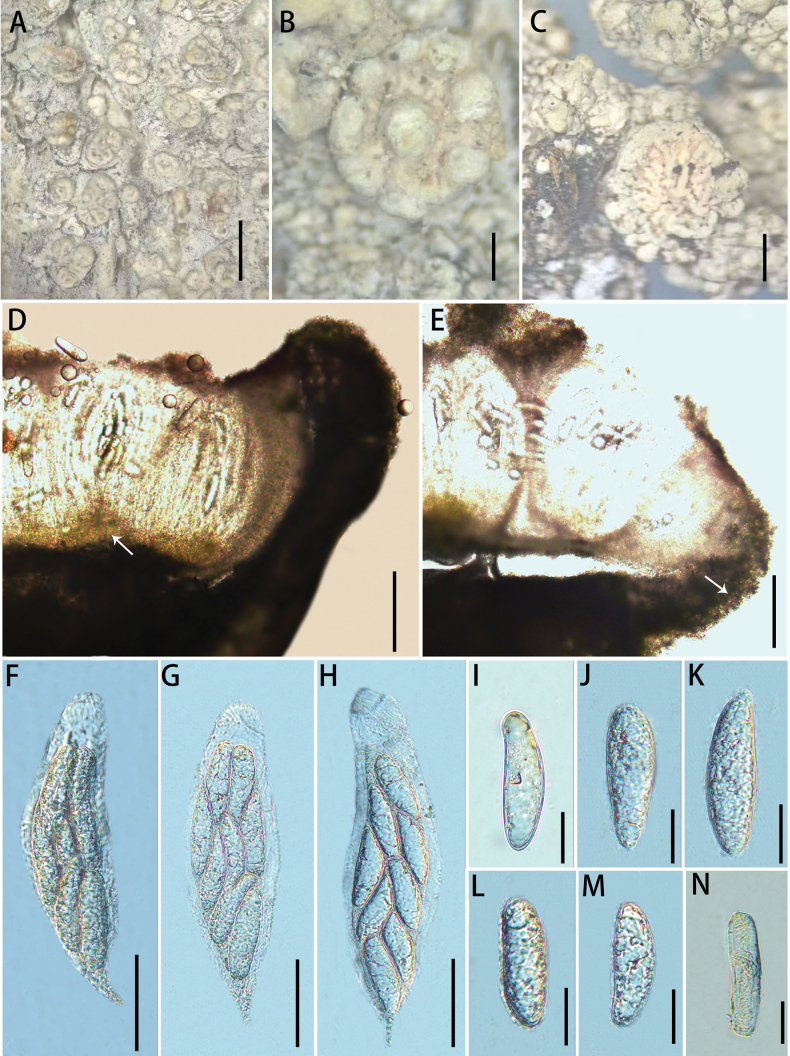
*Ochrolechia kuankuoshuiensis* sp. nov. **A**, **B.** Morphology of thallus and apothecia (KKS83); **C.** Morphology of thallus and apothecia (LGS40); **D**, **E.** Cross-section of an apothecium (the white arrow points to the algae); **F–H.** Asci; **I–N.** ascospores. Scale bars: 3 cm (**A**); 1 mm (**B, C**); 200 μm (**D, E**); 100 μm (**F–H**); 40 μm (**I–N**).

##### Type.

China • Guizhou, Zunyi City, Kuankuoshui Nat. Res, on bark, 1529 m elev., 2023, WW. Zheng and B. Liu, LGS219 (KUN-L 96619, holotype).

##### Etymology.

The species epithet refers to Kuankuoshui, the locality where the type species was collected.

##### Holotype.

KUN-L 96619.

##### Description.

***Thallus*** greyish-white, thick, scaly, dull, verruculose; prothallus indistinct; isidia and soredia absent.

***Sexual morph*. *Apothecia*** frequent, mostly scattered, sometimes crowded, sessile, irregularly rounded or irregularly florid, 0.5–2.5 mm diam.; ***disc*** light yellow, epruinose, rough to rugose when young, with deep rugose when older, margins thick, concolorous with the thallus, shiny, verrucose and higher than disc when young, warts developing until they intersect with the folds of the disc at maturity. Pycnidia are absent. ***Hymenium*** hyaline, 410–483 μm high; paraphyses are branched, densely aggregated; epihymenium black brown (the colour disappears in a 10% KOH solution), 103–125 μm high; hypothecium 50–75 μm high; coccoid green alga, algae forming a continuous layer in the margin and below the hypothecium; ***asci*** clavate, (280)285–309(312) × (66)67–74(75) μm (n = 10), 8-spored. ***Ascospores*** (90)97–116(123) × (30)33–35(36) μm (n = 33), aseptate, hyaline, broadly ellipsoid.

##### Chemistry.

Thallus and ascomata contain grophoric acid and lecanoric acid (TLC). Thallus cortex: K –, C + red, KC + red; medulla: C –; apothecia cortex: K + yellow, C + red, KC + red; medulla: C –; disc: C + red; thallus UV −.

##### Material examined.

China • Guizhou Province, Zunyi City, Kuankuoshui National Nature Reserve, 28°14'14.51"N, 107°9'16.55"E, 1529 m elev., on bark, 17 November, 2023, Weiwei Zheng and Bo Liu, KKS83 (KUN-L 96619, holotype); • Qiandongnan Prefecture City, Leigongshan National Nature Reserve, 26°23'6.73"N, 108°12'11.11"E, 2054 m elev., on bark, 17 October, 2023, Shaobin Fu and Ze Yang, LGS40 (KUN-L 96620).

##### Notes.

Phylogenetic analysis indicates that *O. kuankuoshuiensis* and *O. parellula* are closely related. However, there is a 2.62% (13/496 bp) difference in the ITS sequence between strain KKS83 (*O. kuankuoshuiensis*) and strain KoLRI No.018698 (*O. parellula*). Additionally, *O. parellula* is a saxicolous species characterised by a smooth apothecial margin and smaller spores (40–52.5 × 20–22.5 μm) (Park et al. 2019).

Judging by the description of *O. trochophora* in Brodo (1991), *O. kuankuoshuiensis* is morphologically and chemically similar to this species. However, the latter has smaller spores (35–71 × (20)23–35(–40) μm (Brodo 1991). Sequence comparison between the two species shows an ITS difference of 12.58% (62/493 bp) and an mtSSU difference of 2.99% (21/703 bp) between *O. kuankuoshuiensis* (KKS83) and *O. trochophora* (J. Vondrak 15442). Another morphologically similar species, *O. longispora*, is characterised by a thin thallus, smooth to slightly verrucose apothecial margins and the presence of lichesterinic and protolichesterinic acid (Ren 2017). The ITS sequence difference between *O. kuankuoshuiensis* (KKS83) and *O. longispora* (LGS231-2) is 3.73% (19/509 bp).

#### ﻿ Ochrolechia leigongshanensis

Taxon classificationFungiPertusarialesOchrolechiaceae

Zheng & Fu sp. nov.

BCB6765C-A687-5E9B-83BB-7E81BD969340

Index Fungorum: IF904112

Facesoffungi Number: FoF17960

Fig. 4

##### Remark.

*Ochrolechia leigongshanensis* is distinguished by the presence of soralia and the thallus contains gyrophoric acid, lecanoric acid and atranorin. In most apothecia, a ring of smooth, salmon-pink tissue is visible along the inner edge of the apothecial margin.

**Figure 4.**
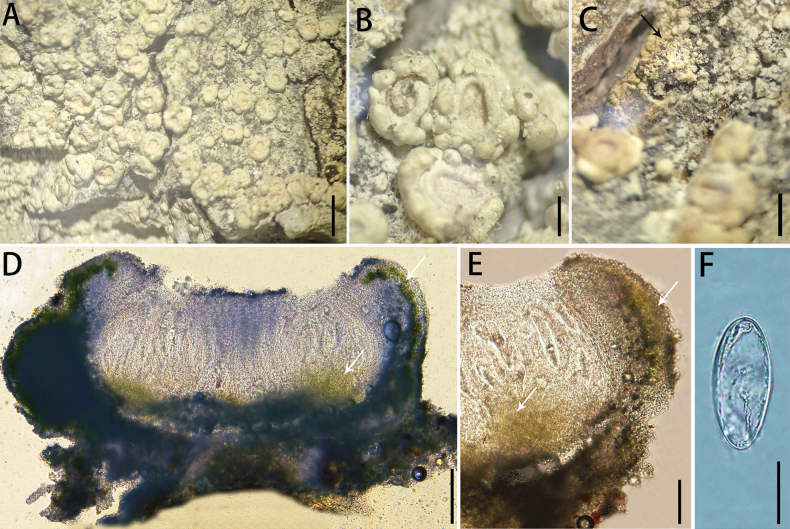
*Ochrolechia leigongshanensis* sp. nov. LGS186. **A.** Morphology of thallus and ascomata; **B.** Morphology of ascomata; **C.** Morphology of soredia (the black arrow points to the soredia); **D, E.** Cross-section of the ascomata in 10% KOH (the white arrow points to the algae); **F.** Ascospores. Scale bars: 5 mm (**A**); 1 mm (**B, C**); 200 μm (**D**); 100 μm (**E)**; 30 μm (**F**).

##### Type.

China • Guizhou, Qiandongnan Prefecture, Leigongshan Nat. Res, on bark, 1666 m elev., 2023, B. Liu and Z. Yang, LGS186 (KUN-L 96621, holotype).

##### Etymology.

The new species has been named after Leigongshan, the locality where it was collected.

##### Holotype.

KUN-L 96621

##### Description.

***Thallus*** greyish-white, thin, verruculose, dull; prothallus indistinct; isidia absent, with a few white soralia.

***Sexual morph*. *Apothecia*** frequent, scattered or crowded, sessile, round, 1.9–2.7 mm diam.; disc plane, pale pink, epruinose to lightly pruinose, margins thick, clearly verrucose, higher than disc, shiny, concolorous with the thallus, a ring of smooth, salmon-pink tissue is distributed along the inner edge of the apothecial margin in most apothecia. Pycnidia are absent. ***Hymenium*** hyaline and colourless, 168–180 μm high; paraphyses are branched, densely aggregated; epihymenium dark brown (the colour disappears in a 10% KOH solution), 10–13 μm high; hypothecium inconspicuous; coccoid green alga, algae forming a continuous layer in the margin and in scattered clumps below the hypothecium. ***Asci*** narrowly clavate, 4–8-spored, 208–230 × 25–33 μm (n = 10). ***Ascospores*** hyaline, broadly ellipsoid, aseptate, (62)64–69(70) × (27)28–33(34) μm (n = 30).

##### Chemistry.

Thallus contains gyrophoric acid and lecanoric acid and atranorin; ascomata contain gyrophoric acid, lecanoric acid (TLC). Thallus cortex: K+ pale yellow, C + red, KC + red; medulla: C –; apothecia cortex: K + yellow-green, C + red, KC + red to yellow-green; medulla: C –; disc: C + red; thallus UV –.

##### Material examined.

China • Guizhou Province, Qiandongnan Prefecture, Leigongshan National Nature Reserve, 26°22'42.92"N, 108°11'42.51"E, 1666 m elev., on bark, 27 October, 2023, Bo Liu and Ze Yang, LGS186 (KUN-L 96621, holotype).

##### Notes.

Phylogenetic analysis places this species in a distinct, but weakly supported clade, sister to *O. akagiensis* and *O. subrosella*. Nucleotide comparison shows that *O. leigongshanensis* (LGS186) differs from *O. akagiensis* (Hara Kojiro: 0007) by 8.07% (39/483 bp) in the ITS region and from *O. subrosella* (LGS176) by 8.49% (41/483 bp) in the ITS sequence and 1.45% (10/688 bp) in the mtSSU sequence. Moreover, both *O. subrosella* and *O. akagiensis* lack soralia and atranorin (Ren 2017) and a ring of salmon-pink tissue is absent from the inner edge of the apothecial margin. *O. akagiensis* produces isidia (Park et al. 2019).

According to the description of O. trochophora var. trochophora in the identification key by Ren (2017), *O. leigongshanensis* is similar to this taxon, but the latter lacks atranorin, without soredia and the excipular ring of salmon disc-like tissue is not visible (Brodo 1991). Judging by the description of *O. montana* in Brodo (1991), *O. leigongshanensis* is also similar to this taxon, but *O. montana* lacks soredia and has smaller apothecia (0.7–1.8 mm in diameter). Chemically, it produces gyrophoric acid and a trace of lecanoric acid as major substances, sometimes along with trace of an unidentified minor compound.

#### ﻿ Ochrolechia longispora

Taxon classificationFungiPertusarialesOchrolechiaceae

Brodo & Q. Ren

AE5BF1BC-411E-566F-B121-0D8B7192DAA3

Fig. 5

##### Remark.

Lichenologist 49: 67–84 (2017); Type: China, Shaanxi, Mt. Taibai, Wengong Temple, on Abies, 3600 m elev., 2005, Q. Ren 1081 (SDNU–holotype).

**Figure 5.**
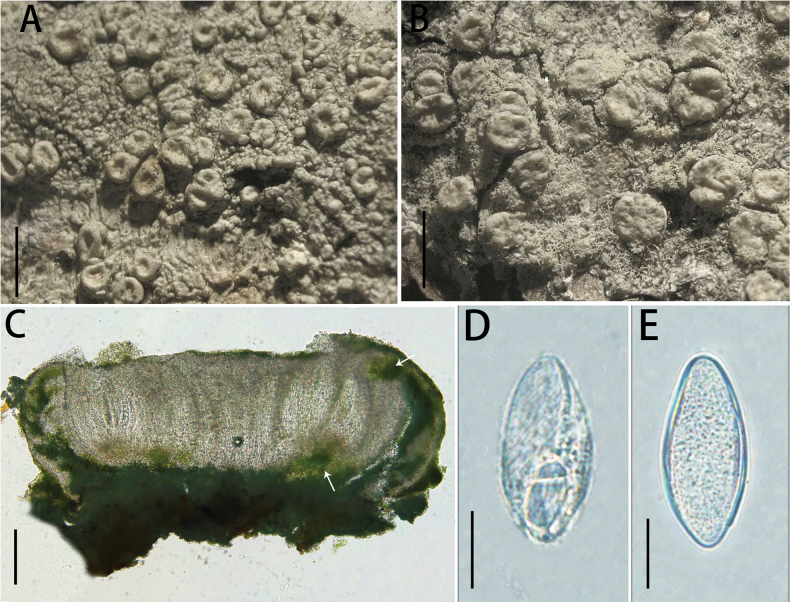
*Ochrolechia longispora* Brodo & Q. Ren. **A.** Morphology of thallus and apothecia (LGS213-2); **B.** Morphology of thallus and apothecia (LGS205); **C.** Cross-section of the ascomata (the white arrow points to the algae); **D, E.** Ascospores. Scale bars: 2 mm (**A, B**); 200 μm (**C**); 30 μm (**D, E**).

##### Description.

***Thallus*** greyish-green, thin to thick, verruculose, dull; prothallus distinct, pale yellow, shiny; isidia and soredia absent.

***Sexual morph*. *Apothecia*** frequent, scattered or crowded, sessile, round, 0.5–1.0 mm diam., disc plane, pale yellow or pinkish, epruinose; margins thick, concolorous with the thallus, higher than disc, smooth or with a few verrucae, shiny. Pycnidia are absent. ***Hymenium*** hyaline and colourless, 340–420 μm high, paraphyses are branched, densely aggregated; ***epihymenium*** pale brown, 40–50 μm high; ***hypothecium*** pale brown (the colour of the epihymenium and hypothecium disappears in a 10% KOH solution), 45–50 μm high, ***algae*** forming a continuous layer in the margin and in scattered clumps below the hypothecium. ***Asci*** narrowly clavate, (230)235–251(255) × (29)31–50 (n = 10) μm. ***Ascospores*** simple, hyaline, aseptate, ellipsoid to broadly ellipsoid, (78)82–89 (91) × (32)35–43(45) μm (n = 30).

##### Chemistry.

Thallus and ascomata contain gyrophoric acid, lecanoric acid, lichesterinic acid and traces of protolichesterinic acid (TLC). Thallus cortex: K + pale yellow, C + red, KC + red; medulla: C –; apothecia cortex: K + pale yellow, C + red, KC + red to yellow-green; medulla: C –; disc: C + red; thallus UV –.

##### Material examined.

China • Guizhou Province, Qiandongnan Prefecture, Leigongshan National Nature Reserve, 26°22'43.41"N, 108°11'42.47"E, 1660 m elev., on bark, 27 October, 2023, Bo Liu and Ze Yang, LGS213-2 (KUN-L 96622); • 26°22'43.17"N, 108°11'42.62"E, 1682 m elev., on bark, 27 October, 2023, Bo Liu and Ze Yang, LGS205 (KUN-L 96623).

##### Notes.

This study provides the first DNA sequences for *Ochrolechia longispora*. *O. longispora* is distinguished by its larger spores and the presence of four specific compounds. Phylogenetic analysis shows that *O. longispora* is closely related to *O. kuankuoshuiensis*; however, we confirm that they are separate species. Since the key differences have already been detailed in the previous section, we will not repeat the comparison here.

According to Ren (2017), *O. longispora* is characterised by a thin, rugose to verruculose thallus; thick, smooth or sparsely verrucose apothecial margins; ascospores measuring 80–90(–105) × 32–42 μm; and the presence of gyrophoric acid, protolichesterinic acid, lichesterinic acid and a trace of lecanoric acid. These characteristics show a high degree of concordance with those of our specimens.

#### ﻿ Ochrolechia subrosella

Taxon classificationFungiPertusarialesOchrolechiaceae

Z.F. Jia & Z.T. Zhao

12DF6B28-5BF9-5AC8-9E50-A5CFD0018FCB

Fig. 6

##### Remark.

Mycosystema 24 (2): 162 (2005); type: China, Yunnan, Dali, Wei WY014-1 (HMAS-L–holotype).

**Figure 6.**
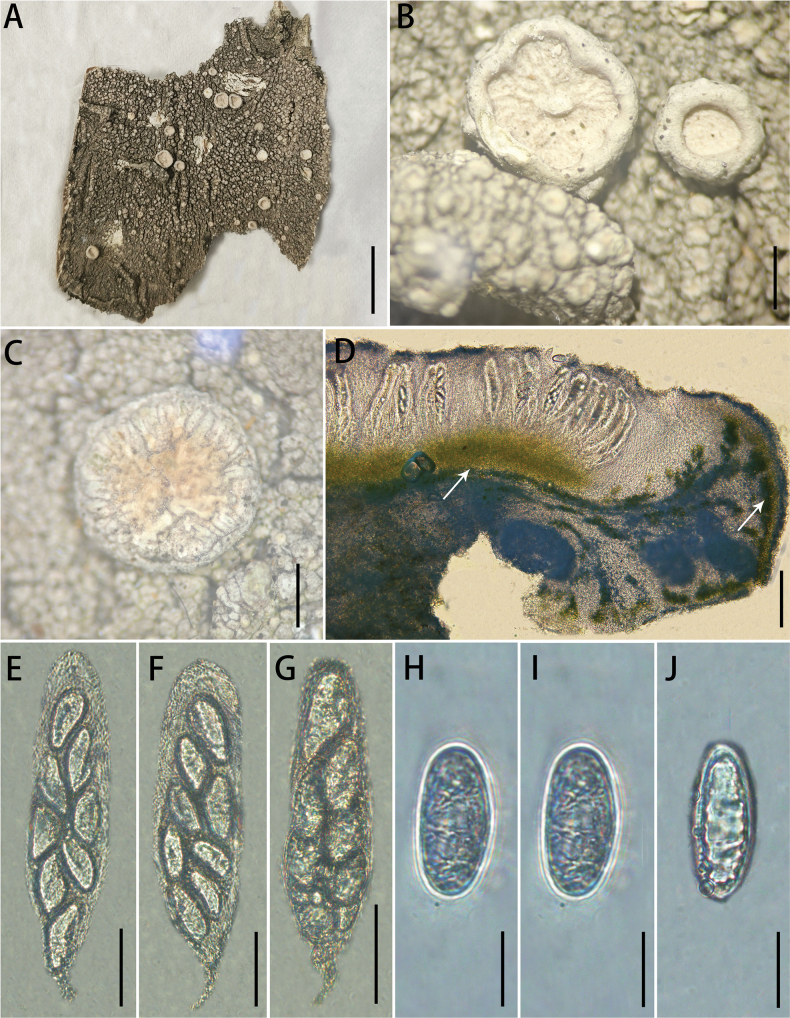
*Ochrolechia subrosella* Z.F. Jia & Z.T. Zhao (LGS176). **A.** Morphology of thallus and apothecia; **B, C.** Morphology of ascomata; **D.** Cross-section of the ascomata in 10% KOH (the white arrow points to the algae); **E–G.** Asci; **H–J.** Ascospores. Scale bars: 1.2 cm (**A**); 3 mm (**B**); 1 mm (**C**); 200 μm (**D**); 60 μm (**E–G**); 30 μm (**H–J**).

##### Description.

***Thallus*** greyish-brown, thick, cracked, verruculose to verrucose, dull; prothallus indistinct; isidia and soredia absent.

***Sexual morph*. *Apothecia*** frequent, scattered, sessile and round, becoming remarkably large at maturity (2.5–4 mm in diameter) and covered with white pruina ***Disc*** pale pink, plane when young, rough to rugose at maturity, followed by a radial bulge; margins thick and smooth when young, becoming thin and verrucose when older, slightly lighter in colour than disc. Pycnidia are absent. ***Hymenium*** hyaline, 290–340 μm high; epihymenium brown (the colour partially or completely disappears in a 10% KOH solution), 20–25 μm high; ***hypothecium*** 20–30 μm high; coccoid green alga, ***algae*** forming a continuous layer in the margin and below the hypothecium. ***Asci*** narrowly clavate, 8-spored, (210)218–243(250) × (51)56–67(70) μm (n = 11). ***Ascospores*** hyaline, aseptate, ellipsoid to broadly ellipsoid, (50)54–64(67) × (22)25–31(33) μm (n = 30).

##### Chemistry.

Thallus and ascomata contain gyrophoric acid and lecanoric acid (TLC). Thallus cortex: K –, C + red, KC + red; medulla: C –; apothecia cortex: K + yellow-green, C + red, KC + red to yellow-green; medulla: C –; disc: C + red; thallus UV –.

##### Material examined.

China • Guizhou Province, Qiandongnan Prefecture City, Leigongshan National Nature Reserve, 26°22'41.82"N, 108°11'43.23"E, 1670 m elev., on bark, 27 October, 2023, Bo Liu and Ze Yang, LGS-176 (KUN-L 96624).

##### Notes.

This study provides the first DNA sequences for *Ochrolechia subrosella*. Phylogenetic analysis shows that this species is closely related to *O. akagiensis* and *O. leigongshanensis*, but forms a distinct, weakly-supported clade. Sequence comparison reveals that *O. subrosella* (LGS176) differs from *O. akagiensis* (Hara Kojiro: 0007) by 6.93% (37/534 bp) in ITS and from *O. leigongshanensis* (LGS186) by 8.49% (41/483 bp) in ITS and 1.45% (10/688 bp) in mtSSU.

Morphologically, this species is characterised by mature apothecia with expanded discs bearing striated protuberances and conspicuous radial ridges, consistent with the original description of *O. subrosella* (Jia and Zhao 2005). Chemically, TLC confirmed the presence of gyrophoric acid and lecanoric acid in LGS176, matching the secondary metabolite profile reported for *O. subrosella*.

#### ﻿ Ochrolechia trochophora (Vain.) Oshio var. trochophora

Taxon classificationFungiPertusarialesOchrolechiaceae

8F464CDC-0A9A-5A00-BEA2-E9E6E6453064

Fig. 7

##### Remark.

J. Sci. Hiroshima Univ., Ser. B, Div. 2(12): 145 (1968). – *Pertusaria trochophora* Vain., Bot. Mag. (Tokyo) 32: 155 (1918).

**Figure 7.**
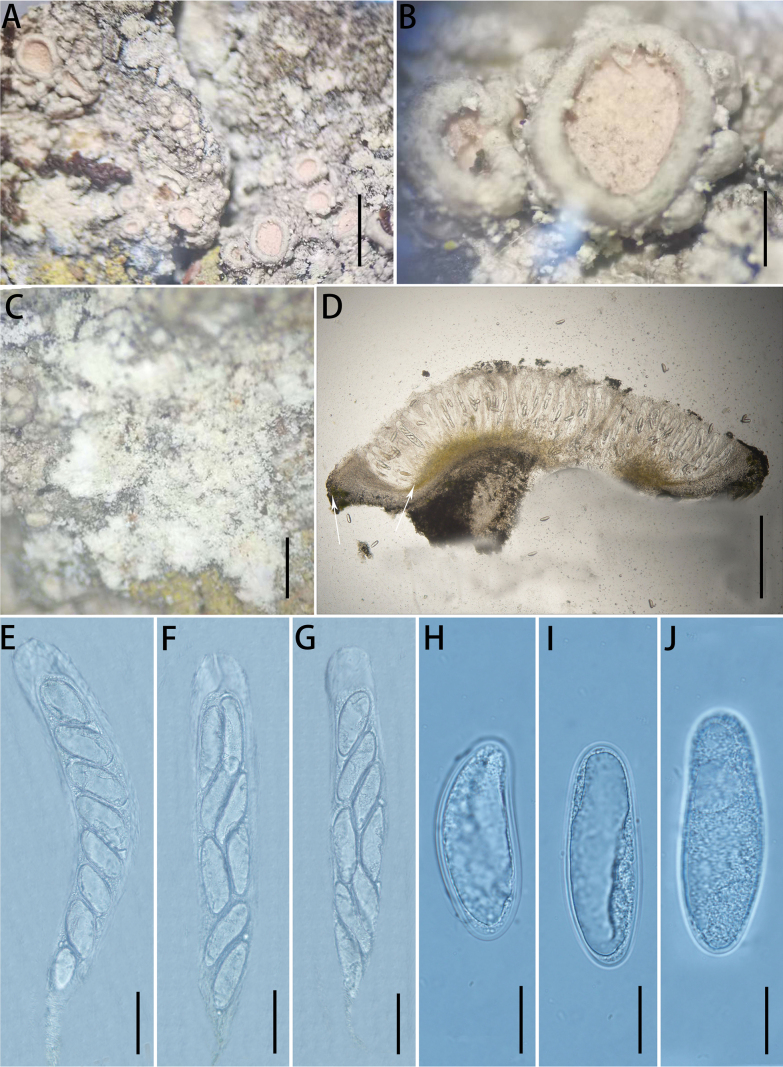
Ochrolechia trochophora (Vain.) Oshio var. trochophora, J. Sci. Hiroshima Univ (LGS150-2). **A.** Morphology of thallus and apothecia; **B, C.** Morphology of ascomata; **D.** Cross-section of the ascomata in 10% KOH (the white arrow points to the algae); **E–G.** Asci; **H–J.** Ascospores. Scale bars: 3 mm (**A**); 0.7 mm (**B**); 1 mm (**C**); 500 μm (**D**); 50 μm (**E–G**); 20 μm (**H–J**).

##### Type.

Japan • Prov. Kozuke, Mt. Akagi. Ad corticem arboris, Yasuda 53 (TUR-V–7255 – holotype, not seen; see Brodo (1991: 762); TI – isotype, not seen; see (Oshio 1968: 145)).

##### Description.

***Thallus*** greyish-white to greyish-green, thin, verruculose, dull; isidia absent, prothallus indistinct, soredia greyish-white.

***Sexual morph*. *Apothecia*** frequent, scattered, sessile, ellipsoid or round, 1.1–1.8 mm diam.; disc pale pink, plane to lightly rugose, epruinose, with a hazy white film; margin smooth or verruculose, higher than disc, dull, concolorous with the thallus. Pycnidia are absent. ***Hymenium*** hyaline and colourless, 380–392 μm high; epihymenium brown, 20–28 μm high; hypothecium greyish-brown (the colour of the epihymenium and hypothecium partially or completely disappears in a 10% KOH solution), 10–17 μm high; coccoid green alga, algae absent or spotty in the margin and continuous below the hypothecium. ***Asci*** clavate, 8-spored, (359)363–372(375) × (42)43–49(52) μm. ***Ascospores*** hyaline, aseptate, broadly ellipsoid, (50)55–70(75) × (22)23–26(28) μm (n = 30).

##### Chemistry.

Thallus contains gyrophoric acid, lecanoric acid and atranorin; ascomata contain gyrophoric acid and lecanoric acid. (TLC). soredia: K+yellow-green, C –, KC –; thallus cortex: K + yellow, C + red, KC + red to yellow-green; medulla: C –; apothecia cortex: K + yellow, C + red, KC + red to yellow-green; medulla: C –; disc: C + red; thallus UV –.

##### Material examined.

China • Guizhou Province, Zunyi City, Kuankoshui National Nature Reserve, 28°12'29.34"N, 107°10'24.27"E, 1418 m elev., on bark, 18 November, 2023, Weiwei Zheng and Bo Liu, KKS150-2 (KUN-L96625).

##### Notes.

*Ochrolechia trochophora* is a widely distributed species reported from several locations worldwide. It is primarily characterised by verruculose apothecial margins lacking or containing scattered algal cells. The key distinction between O. trochophora var. trochophora and O. trochophora var. pruinirosella lies in the fact that the latter has a pruinose apothecial disc and the vast majority of its specimens contain variolaric acid. In contrast, O. trochophora var. trochophora occasionally exhibits a hazy white film on the disc, but this is not pruina and very few specimens contain variolaric acid or atranorin (Brodo 1991). As reported by Kukwa (2009), European and Turkish specimens of O. trochophora var. trochophora possess a relatively thin thallus. Furthermore, the detection of atranorin in one Chinese specimen aligns with these findings. Collectively, this evidence supports the identification of specimen KKS150-2 as O. trochophora var. trochophora.

The specimen also bears soredia, which is a relatively distinctive variation, as according to previous reports, no records of soredia have been documented in *O. trochophora* (whether the typical variety or other known varieties) (Brodo 1991; Kukwa 2009). This indicates that *O. trochophora* might be morphologically more variable than previously thought.

In *Ochrolechia*, many species (e.g. *O. africana*, *O. antillarum*, *O. gowardii*, *O. trochophora*) contain small crystalline structures in the apothecial medulla (Brodo 1991). In the species we describe, the brownish appearance of both the epihymenium and hypothecium in apothecial sections is more likely a structural colour rather than a pigment-based one. This conclusion is primarily based on the partial or complete fading of the brown colour when treated with 10% KOH, likely due to the dissolution of abundant granular or crystalline substances in the tissues.

Traditionally, the classification of species within the genus *Ochrolechia* has primarily relied on a combined analysis of morphological characteristics and chemical compounds. Morphologically, key diagnostic features include thallus thickness, the presence or absence of isidia and soredia, apothecial morphology, hymenium height, spore size and the position of the algal layer. Chemically, species in this genus exhibit a remarkable diversity of secondary metabolites, which serve as critical taxonomic markers. These compounds mainly belong to the following classes: Orcinol depsides (e.g. gyrophoric acid, lecanoric acid and olivetoric acid), orcinol depsidones (e.g. variolaric acid and alectoronic acid), higher aliphatic acids (e.g. lichesterinic acids, protolichesterinic acids and murolic acids) and xanthones, which can induce yellow fluorescence in the thallus under long-wave ultraviolet light. Additionally, trace amounts of atranorin have been detected in some species (Brodo 1991; Kukwa 2009).

In our study, thin-layer chromatography revealed significant levels of the lichen secondary metabolite atranorin in both the newly-described species *O. leigongshanensis* and the known species O. trochophora var. trochophora. This finding contrasts with the previous understanding that atranorin exists only in trace amounts within the genus *Ochrolechia*. Notably, these two species share a key morphological characteristic, the presence of soredia. The combination of this chemical and morphological feature provides valuable new insights into the evolutionary relationships within *Ochrolechia*.

Soredia are rarely observed in *Ochrolechia* and atranorin is not usually a major metabolite in this genus. The discovery that both *O. leigongshanensis* and O. trochophora var. trochophora possess these two characteristics strongly suggests that they may belong to a distinct phylogenetic lineage previously unrecognised. The production of soredia, as an asexual reproductive structure, is linked to specific genotypes, while the substantial synthesis of atranorin indicates the activation of particular biochemical pathways. The stable co-existence of these two independent traits, both morphological and chemical, within a limited taxonomic group is unlikely to be coincidental and likely represents synapomorphies inherited from a common ancestor. Thus, we hypothesise that these two species may share a most recent common ancestor within *Ochrolechia*, potentially forming a monophyletic group.

With the rapid advancement of molecular techniques, phylogenetic analysis has become an indispensable tool in species identification and evolutionary studies. Molecular data provide objective genetic evidence that enables the effective differentiation of morphologically similar and cryptic species, clarifying taxonomic uncertainties, such as synonymy and facilitating the discovery of new taxa (Spatafora et al. 2006; Miadlikowska et al. 2014). Relying solely on phenotypic characteristics often fails to resolve taxonomic ambiguities, as these features are influenced by environmental factors and developmental stages. In contrast, genetic data offer greater stability. Therefore, this study adopts an integrative taxonomic approach, combining phylogenetic analysis, morphological examination and chemical profiling to provide a comprehensive and accurate classification of new *Ochrolechia* species.

The phylogenetic analysis in this study revealed that all newly-described species form a single clade (Fig. 1). Although bootstrap support for some branches within this clade was relatively low (< 70%), we interpret this not to ambiguous taxon delimitation, but rather to the substantial genetic distances amongst these new species or it might be due to the insufficient sampling in this clade. As more sequences of *Ochrolechia* species are published in the future, the topology may become more stable. Such considerable interspecific genetic divergence may result in a higher number of ambiguous alignment sites, leading to reduced nodal support in phylogenetic reconstructions. This phenomenon strongly suggests that the present study may have only uncovered a fraction of the diversity within the genus *Ochrolechia* in Guizhou, China. Limitations in sampling scope and specimen numbers currently hinder a full assessment of the true morphological variation and geographical distribution ranges of these species, which may also contribute to the temporarily unresolved phylogenetic relationships.

It is important to note that, while molecular support could be further strengthened through additional gene loci or expanded sampling in the future, all new species described in this study exhibit unique combinations of morphological and chemical characteristics, enabling clear distinction from all known related species.

In conclusion, the distinct clade formed by the new species described in this study not only enhances our understanding of the phylogenetic framework of *Ochrolechia*, but also highlights significant genetic variation within the clade, suggesting that the region may harbour an underexplored diversity of *Ochrolechia*. Future studies should prioritise more extensive and systematic specimen collection from this region and neighbouring areas, combined with multi-locus genomic data, to thoroughly elucidate the speciation mechanisms and the broader diversity of this genus.

## Supplementary Material

XML Treatment for Ochrolechia guizhouensis

XML Treatment for Ochrolechia kuankuoshuiensis

XML Treatment for Ochrolechia leigongshanensis

XML Treatment for Ochrolechia longispora

XML Treatment for Ochrolechia subrosella

XML Treatment for Ochrolechia trochophora (Vain.) Oshio var. trochophora

## ﻿Additional information

### Use of AI

No use of AI was reported.

### Author ORCIDs

Weiwei Zheng https://orcid.org/0009-0006-4099-7599

Linzhi He https://orcid.org/0009-0008-3621-7339

Heyun Bo https://orcid.org/0009-0003-8641-1619

Rajesh Jeewon https://orcid.org/0000-0002-8563-957X

Ruvishika S. Jayawardena https://orcid.org/0000-0001-7702-4885

Yuxian Wang https://orcid.org/0009-0004-8810-3641

Jie Wang https://orcid.org/0009-0000-7788-4471

Shaobin Fu https://orcid.org/0000-0001-9932-1346

Qingfeng Meng https://orcid.org/0000-0001-9814-8238

### Data availability

All of the data that support the findings of this study are available in the main text.
